# Click me…! The influence of clickbait on user engagement in social media and the role of digital nudging

**DOI:** 10.1371/journal.pone.0266743

**Published:** 2022-06-29

**Authors:** Anna-Katharina Jung, Stefan Stieglitz, Tobias Kissmer, Milad Mirbabaie, Tobias Kroll

**Affiliations:** 1 Department of Digital Communication and Transformation, University of Duisburg-Essen, Duisburg, Germany; 2 Department of Information Systems and Digital Society, Paderborn University, Paderborn, Germany; West Pomeranian University of Technology, POLAND

## Abstract

Clickbait to make people click on a linked article is commonly used on social media. We analyze the impact of clickbait on user interaction on Facebook in the form of liking, sharing and commenting. For this, we use a data set of more than 4,400 Facebook posts from 10 different news sources to analyze how clickbait in post headlines and in post text influences user engagement. The results of our study revealed that certain features (e.g., unusual punctuation and common clickbait phrases) increase user interaction, whereas others decrease engagement with Facebook posts. We further use our results to discuss the potential role of digital nudging in the context of clickbait. Our results contribute to understanding and making use of the effect of different framings in social media.

## 1. Introduction

Social media as a platform for information consumption and distribution has become increasingly important as people switch from traditional media like television or newspapers to online and mobile media as their main media source [1–3]. Youth in particular use social media to stay informed and connected [4–7]. Although people older than 35 years prefer to consume their news through news apps and directly on news websites, those younger than 35 years prefer social media and messengers to stay informed [8]. To stabilize or increase their readership levels to stay profitable, most media organizations jumped on the bandwagon and are present on social media. In 2009, most U.S. newspapers had only a negligible number of followers on social media, but by 2013, all U.S. newspapers with a weekday circulation of more than 100,000 used Facebook or Twitter [9]. Due to the reluctance of media users to pay for online news offers and increasing competition with additional paid entertainment offers such as Netflix and Amazon Prime, media organizations face pressure to attract readers to their websites [8,10,11] to generate income from the displayed advertisements [12].

In addition to the need for clicks, journalists are fulfilling the role of a watchdog, which keeps an eye on those in power, ranging from the government to the economy [13]. This role—known as the fourth estate—did not decrease in relevance due to the digitalization of the media landscape [14]. In times of fake news, which are defined as news articles which intentionally spread deceptive information, this democratic role of journalism has gained importance [15–17]. However, the dynamics resulting from the digitalization and the increasing relevance of social media is an ongoing challenge for quality journalism. Two characteristics of this development are the decreasing attention span of internet users and a decreasing interest in hard news [18,19]. As a reaction to this development, quality outlets have adopted characteristics of the tabloid press, which is referred to as *tabloidization* in the media theoretical context [20].

An effective strategy for drawing attention and capturing readers’ curiosity is clickbait which has risen in recent years and has been adopted by publishers of all kinds especially on social media platforms like Facebook and Twitter [21]. Clickbait has mostly been associated with the tabloid press, for example, “The 10 reasons why everyone should drink coffee” (*Daily Mail*, [22]) but can also be found in quality sources: “A woman had stomach pains. Doctors discovered it was something she swallowed–a decade ago” (*The Washington Post*, [23]). Clickbait is defined as short teaser messages that make use of cognitive biases to increase the likelihood that users click on the accompanying link [11]. Users get the feeling of needing to know the information which can be reached only by clicking on the link [11]. In scientific and societal discourse, clickbait strategies are consistently presented as morally reprehensible actions, which lead to disappointment among the audience, as the media products do not deliver on their promises. Therefore, clickbait as a traffic-generating technique, is rejected categorically by many journalists. Professional journalists feel committed to comply with journalistic codes of conduct, such as the Society of Professional Journalists’ code of ethics [24]. However, first studies found no statistical significant effect between the usage of clickbait features and the media perception of users [25].

Besides the controversial discussions around it, there is still a research gap regarding clickbait. In addition to its ability to attract users’ attention, clickbait can trigger increased user engagement which has become another dimension of social media. User engagement is “*[…] a quality of user experiences with technology that is characterized by challenge*, *aesthetic and sensory appeal*, *feedback*, *novelty*, *interactivity*, *perceived controland time*, *awareness*, *motivation*, *interest*, *and affect*.” [26]. The first studies showed that clickbait features do not activate clicks and increase user engagement to the same extent [27]. Due to this research gap concerning user engagement, a clear statement about clickbait’s efficacy cannot be made. Therefore, the research question of this study is as follows:

### 1.1 RQ: How do clickbait features in news posts with links on Facebook influence user engagement in the form of sharing, commenting and reacting?

To answer the research question, we collected about 4,000 posts from seven consecutive days in late 2017 from 10 U.S. and UK news Facebook pages, including reputable (e.g., *New York Times*) as well as so-called tabloid news sources (e.g., *Daily Mail*). We chose the social network Facebook as it was the most important social network for news consumption when the study was conducted [8]. With a negative binomial regression analysis, we calculate the isolated effects of different features and controls on count-variable shares, comments and reactions (e.g., likes). Accordingly, we test the impact of previously identified clickbait features and provide new insights into user engagement on Facebook.

In addition to answering this central research question, we want to draw additional attention to potentially “good” use of clickbait and bring into play what behavioral economists have called nudge theory [28] and has been proposed for digital environments [29,30]. To discuss the role of digital nudging in the context of clickbait, we review literature on the principle that clickbait strategies are potentially morally reprehensible and on the previously mentioned *tabloidization* of hard news. With the findings from the social media posts, we contribute by discussing the nudge perspective on clickbait.

The paper is concluded by a summary of theoretical and practical contributions.

## 2. Related work

### 2.1 Social media for news and the role of clickbait

Social media have changed the media landscape as not only journalists but also all internet users are able to participate in the process of producing and disseminating news [31,32]. The abundance of information available, especially on social media, can give users the illusion of knowing, which describes the mismatch between what users think they know and what they actually know and comprehend [33,34]. Often, users do not read the entire online article, do not check the veracity of the sources and do not look for further information on the topic [4,35]. In recent years, media organizations have been integrating social media into their daily routines, as tools for sourcing, disseminating, curating and discussing news stories [36,37]. Media organizations monitor their social media impact increasingly closely, which affects their editorial decisions [38,39].

Clickbait is an editorial technique of writing headlines and snippets that direct users to websites. This might violate journalistic codes of conduct, such as the Society of Professional Journalists’ (SPJ) Code of Ethics, for several reasons [11,24]. The four principles of the SPJ Codes of Ethics are exemplary for many other journalistic codes: “Seek Truth and Report It, Minimize Harm, Act Independently, Be Accountable and Transparent.” As the use of clickbait often occurs in relation to advertisements and incomplete or even false information, clickbait often collides with the principles of Independence and Truth [40]. Especially the usage of clickbait features in the context of high-quality journalism must be treated with caution. Current state of literate suggests a mixed view on the usage of clickbait in the context of quality content. Generally, e.g., Muddiman and Scacco [41] mention the effect of a negative perception of otherwise quality imaged media sources. Adding to this in a more holistic view, a meta study [25] summarizes that the negative effect is assumed, yet research lacks proof of this. Studies evaluating this mostly had non-significant results or tendencies as conclusion. “*Clickbait headlines*, *on the other hand*, *appear to have broadly negative impact on audience perceptions*, *though the effects are small*.*”* [25]. Yet, even thought the definite negative impact is not widely proven in research, we need to assume interferences. This can lead to the mix of quality and tabloid content, making it hard for users to differentiate the source’s quality.

This in term can have multiple consequences. The main purpose news sources to inform users is harmed substantially if users loose trust in the source. The trust in a source is not always formed by the news content itself but sometimes through other external factors [25]. With this, the presentation of the content itself also counts to these factors, making it an influencer for the formation of trust. The latter converts to loyal customers and hence to more active readers. This gain of credibility is essential for quality journalism [42]. It can be deducted that without the necessary credibility, an information of readers if ineffective or prone to decease. Studies found that credibility is mainly defined by the quality of the news as such, and the experience with similar content [25,43]. With a skeptical view on clickbait, the user’s experience with tabloid content might jump over to high-quality journalism that is presented in a similar way using clickbait technologies.

However, current findings lead to the assumption that categorical normative rejection of traffic-generating techniques by journalists does not fully coincide with reception by the audience. As a reaction to this development, quality outlets have adopted characteristics of the tabloid press, which is referred to as *tabloidization* in the media theoretical context [20]. Just as research indicates the potential issues with clickbait in relation to quality journalism, other findings contradict and see the influence of clickbait subordinated. The study by Johnson and Kaye [43] found that the credibility for news sources increases if the content is easily accessible or presented in a convenient way. Mirroring this to clickbait, the simplification and usage of standard clickbait phrases is assumed to be more easily consumable and thus a positive effect on credibility is possible. This is supported by a longitudinal study that assigns the easily consumable content an increased trust [44]. The assumption that this is due to the more frequent usage and thus a certain accommodation or habit, is yet to be validated. Besides this, Fico et al. [45] assigned the generation of credibility for news sources mainly to the stories being balanced and of high quality. Contradicting this, a study found that news content provided by the European Union does not generate new readers through higher content quality but rather through the use of catchy phrases and thus the simplification of presentation [46]. Furthermore, a standard developed over years and users are more and more used to clickbait, making the handling of it common-sense and potentially remediating the negative effect [47]. In a nutshell, the usage of clickbait in quality journalism is controversially discussed in many studies. We found many indicators for a positive influence on the user’s perception but also many pointers towards a potential negative influence, harming the source’s credibility. A careful handling of the topic is hence advisable.

Despite the ethical doubts, even high-prestige news platforms consider tabloidization to capture readers’ curiosity, including the creation of new soft news sections and the increasing application of commercialization and tabloidization with clickbait in the formulation of their headlines [48,49]. Soft news deal with lifestyle and entertainment, for example, and are shared more often than hard news, such as politics and economics [18]. Tabloidization is understood as “the convergence of ‘quality’ newspapers towards the values characteristic of tabloid newspapers, which can be identified as personalization and sensationalism, and the linguistic features through which these values are represented” [50]. Summarizing, online news now mimics successful platforms that use clickbait and similar methods to lure users to their websites.

Clickbait uses narrative and stylistic devices to attract the reader’s attention [49] which can misinform readers if they do not read the full story [10]. Clickbait can also take place in non-text cues with pictures that attract attention before headlines or other text is read [40,51]. However, many media organizations apply it which can be linked to the increasing competitive pressure and their financial dependence on traffic and clicks to their websites [12].

### 2.2 Clickbait strategies and features

Previous research on clickbait primarily focused on article headlines [52]. The writing style of the headlines of reputable newspapers and tabloid newspapers differs, and thus, they are perceived differently during the information-seeking process [53]. Scacco and Muddiman (2019) described the headlines of reputable newspapers as “summary headlines,” which clearly state the main information of an article in the headline. In contrast, the authors described tabloid headlines which use clickbait features as “curiosity headlines,” which aim at encouraging audience engagement, for example, with emotionally loaded words [54,55]. A study on the tabloid *Daily Mail*’s most read articles found common linguistic features (e.g., sensationalism) used to present an issue as more relevant and extraordinary [56]. In an attempt to build a browser extension that automatically detects and blocks clickbait headlines, Chakraborty et al. (2016) identified features that occur more frequently in clickbait headlines than in non-clickbait headlines: The sentence structure differs, and clickbait headlines contain more words, but the words are shorter. Regarding the use of punctuation, clickbait headlines show patterns of several marks in a row (e.g., …,!?, !!!). They comprise more questions as they should activate the reader [52]. Certain words or phrases in headlines (e.g., “you won’t believe”) have been identified as another indicator of clickbait [52,57]. Stop words and hyperbolic words that express highly positive sentiments (e.g., “best ever”) are frequently used [58]. They all arouse curiosity in the reader by implying that the content of the article (which remains nebulous) is exceptional or shocking. In addition to longer phrases, Biyani et al. (2016) identified a series of unigrams (i.e., an isolated word like *video*) and bigrams (i.e., two isolated serial words like *watch video*) often used in the title and body of a clickbait article.

### 2.3 Nudge theory and its relation to clickbait

The efficacy of clickbait that lets people click or interact has been explained and improved with cognitive theories such as the Loewenstein information-gap theory of curiosity [11], and can be related to dual process theories, like the elaboration likelihood model [59]. In essence, such theories suggest different modes of cognitive processing that run either automatically or consciously. Clickbait makes use of peripheral cues that do not require much cognitive capacity but trigger arousal [12]. Although the connotations and the applications of clickbait have usually been negative, a related theory from behavioral economics suggests a positive application.

The Nudge theory defines nudges as “any aspect of the choice architecture that alters people’s behavior in a predictable way without forbidding any options or significantly changing their economic incentives” [28]. A nudge is comparable to clickbait as the choice architecture is changed in a way that encourages a click. At the same time, a nudge differs from clickbait as Sunstein and Thaler also stressed the goal of nudging as making “lives longer, healthier and better” (p. 5), that is, having a positive effect on the individual instead of clicks for an increased revenue. That also distinguishes nudging from pure persuasion and its research stream in human–computer interaction (HCI) which does not necessarily require a noble goal [60,61].

With digital nudging, the idea of using or reducing decision biases to guide people toward better behavior has been introduced to the digital sphere [29]. Combining persuasion and nudging literature, digital nudging is defined “as a subtle form of using design, information and interaction elements to guide user behavior in digital environments, without restricting the individual’s freedom of choice” [60]. Digital nudges have been investigated in several contexts (Amojo und Meske 2020), such as health applications [62], privacy awareness [63], online security [64,65], ethics [66] and e-commerce [67]. In addition, scholars have investigated how a browser extension can help users make conscious decisions regarding news credibility on Twitter [68]: The extension FeedReflect changed the appearance of non-mainstream news sources by dimming those posts. At the same time, the study aimed at motivating users to interact with comments of mainstream sources’ posts (including reputable and tabloid news sources). The browser extension was used by 16 students in a three-week study with a significantly better credibility assessment compared to a control group that did not receive any nudges. A follow-up interview with the participants supported the approach to make users rethink the news [68]. However, a browser extension can be only a first approach in research as it requires users to install something and thus, be aware of a potential unwanted behavior. It is questionable if all users would have such a high degree of critical self-reflection about their news consumption habits. The results can be used by social media platform providers, but given their reluctance to implement mechanisms against proper privacy protection, hate speech or fake news [69], it is questionable whether providers would support users in credibility assessments as suggested with FeedReflect. Except users and platform providers, we were not able to find any application of nudging for news sources and social media publishers. Despite the knowledge of clickbait that makes use of cognitive processes similar to nudging, the application of positive digital nudging by news sources on social media has not been studied. In the realm of nudging, ideas and methods for influencing decision-making have also been misused for marketing purposes, for example. Thaler [70] called those applications “sludge” to make clear that a nudge requires noble goals and must (at least indirectly) help the user. As an analogy, clickbait in its prevalent meaning is sludge, while there can also be a nudge equivalent that uses the same or similar methods as clickbait and is the subject of this study. In times of fake news and information overload, reputable journalists could use such methods but for good, that is, to inform people about socially relevant topics and to reduce the spread of false information by making the reputable press more visible to users.

## 3. Motivation and hypothesis development

Originating in tabloid press and soft news, clickbait has become a common occurrence on social media for all kinds of news and outlets [11,12]. An increasing number of journalists consider clickbait to be a useful strategy to attract a larger audience [10], while at the same time they are obligated to preserve objectivity [40,71].

Clickbait is used to tempt users to click on a link and has been deemed an effective instrument for drawing attention to an article [11,49]. Although the click and visit to the website are the goal, such data is not easily accessible or necessarily a sufficient indicator for reading of articles, either [4,35]. In this study, we are interested in the interaction expressed by the numbers of reactions, shares and comments. Although interaction is a different dimension, it is as interesting as clicks because users sometimes interact based on a post and a preview without reading the article [4] or interact actively with a post, after they read the article, as a sign of their satisfaction with the content [27]. Interaction counts are also worth study as they enhance the algorithmic diffusion of a post. Facebook announced in 2018 that the algorithm would prioritize content that is expected to be of interest for the individual user and that would lead to either discussions in the comments or shares and reactions. Facebook pages whose content is rarely interacted with would notice a decrease in distribution [72].

The majority of previous studies on clickbait investigated the impact of headlines and their clickbait characteristics. Chakraborty et al. (2016) [58] identified unusual punctuation marks as a clickbait feature. Likewise, the presence of questions has been associated with clickbait [52]. For example, Al Nuaim et al. (2017) found that the subjectivity of a headline or the article itself leads to more shares on social media platforms. Commenting on a post can also be seen as a way of communicating and sharing one’s opinion with others. Regarding reactions on Facebook (i.e., Like, Sad, Haha, Wow, and Angry), a study showed that post characteristics (e.g., the topic or the presence of pictures) have an impact on the reaction counts [73]. Thus far, researchers have paid little attention to the role of the post text. However, the post text offers authors the possibility to add further and different information to their post than they would usually in an article’s headline. Therefore, we also examine the role of clickbait features in post texts.

In addition to the mentioned clickbait features, we include the sentiment of the text into our analysis as it is found to be a significant influencer for the clickbait success [11]. Studies find that clickbait posts are usually more positively toned, which is causing more user engagement [11,74,75].

Informed by studies that aimed either to classify articles into clickbait and non-clickbait [57] or measured the effectiveness of specific clickbait strategies for the click-through rate [52], we hypothesize that clickbait features in post texts as well as preview headlines influence (a) the number of reactions, (b) the number of shares and (c) the number of comments. The included features and their hypothesized direction are depicted in Table 1. The effect of the listed features is hypothesized for headlines and post texts.

**Table 1:** Hypothesized clickbait features in headline and post text.

Feature	Direction	Reasoning	Source
Average word length	Negative	Readers tend to skip longer words because they require more cognitive capacity.	Chakraborty et al., 2016 [58]; Kuiken et al., 2017 [52]
Number of words	Positive	Too few words cannot convey sufficient information. Longer headlines may also attract attention because they require more space.	Biyani et al., 2016 [57]; Chakraborty et al., 2016 [58]; Kuiken et al., 2017 [52]
Contains questions	Positive	Questions addressing the reader may trigger the need to react or answer.	Kuiken et al., 2017 [52]
Unusual punctuation	Positive	Unusual punctuation (e.g.,!!!) attracts the reader’s attention. It may be perceived as being of higher importance.	Biyani et al., 2016 [57]; Chakraborty et al., 2016 [58]
Typical clickbait phrases (n-grams)	Positive	Typical clickbait phrases like “will blow your mind” stimulate readers and excite their curiosity.	Chakraborty et al., 2016 [58]
Unigrams	Positive	In addition to longer phrases, specific unigrams are often present in clickbait headlines (e.g., video, reason, secret).	Biyani et al., 2016 [57]
Sentiment	Positive	A positive tone of the message is going to increase user engagement.	Chakraborty et al., 2017 [74]

## 4. Research design

### 4.1 Data set

The data was extracted with custom tracking software (Java-based) which connected to the official Facebook Application Programming Interface (API) and stored data of pre-defined Facebook pages in a database. The tracker regularly queried the API for new posts from the defined pages and stored the received data in a database. Before extraction, we made sure that the usage of the API complies with Facebook’s terms and conditions and this is allowed for scientific purposes, which was the case (December 2017). Furthermore, no individual user data was collected in this process, solely the names of the publishing houses were visible for the researchers. The first selection criterion for the pages was a frequently used Facebook page; this included the regular posting of articles (a minimum of 10 per day) and a follower count larger than one million followers to receive sufficient interaction data (e.g., comments, likes). Further, we limited the selection to sources published in English due to the ability of dictionaries or lists, for example, for common clickbait phrases. This allowed us to include sources from the United States and the United Kingdom that are internationally known. We did not limit the topics or types of posts in any further way.

We decided to include news sources from five reputable and five tabloid news sources (see Table 2) to cover a wide range of clickbait (non-)usage [49]. Ten Facebook pages appeared feasible in terms of the expected data set size but large enough for robust analyses. Finally, the data was collected over a period of seven days from 2017 November 27 to 2017 December 3. To ensure that the interaction counts were stable and comparable, these numbers were stored one week after the post was published.

**Table 2:** News sources.

Category	Name	Country	Number of posts	Page ID
Tabloid	*Daily Mail*	UK	676	164305410295882
	*Daily Mirror*	UK	614	6149699161
	*HuffPost*	USA	439	18468761129
	*New York Post*	USA	329	134486075205
	*TIME*	USA	169	10606591490
Reputable	*Guardian*	UK	360	10513336322
	*NY Times*	USA	446	5281959998
	*The Telegraph*	UK	478	143666524748
	*Wall Street Journal*	USA	464	8304333127
	*Washington Post*	USA	436	6250307292

In addition to the publishing time, post ID and post text, we queried the number of reactions, shares and comments seven days after the post was published. This data was stored in an aggregated and thus anonymized way, prohibiting any processing of personal data. The data set contained 4,410 Facebook posts. *TIME* had the lowest number with 169 posts; the *Daily Mail* published the most with 676 posts.

### 4.2 Feature annotation

The analysis of the proposed hypotheses required us to annotate the collected data so that for each post the information regarding use of, for example, unusual punctuation was available. We used Python to process the data with dictionaries and count rules (Table 3). For calculating the number of words and the average word length, we removed special characters and punctuation. To assess the presence of clickbait phrases, we used the list from the Downworthy browser plugin [76]. We used a top nine list containing clickbait unigrams of headlines for the unigram feature [57]. The latter two features as well as the punctuation feature were treated as Boolean variables (present or non-present), and the other three as numerical variables. The six depicted features were separately calculated for the post text and the headline resulting in 12 variables.

**Table 3:** Operationalization of clickbait features.

Feature	Rule/calculation (excerpt)	Type	Source
Average word length	Characters divided by word count	Number	Kuiken et al., 2017 [52]
Number of words	Word count	Number	Kuiken et al., 2017 [52]
Containing questions	Number of single question marks	Number	Biyani et al., 2016 [57]
Unusual punctuation	!!! /!? / … / ***	Boolean	Chakraborty et al., 2016 [58]
Typical clickbait phrases (n-grams)	can change your life, breathtaking *Total*: *119 phrases*	Boolean	Chakraborty et al., 2016 [58]; Gianotto, 2016
Unigrams	“video”/ “photo” / “reason”	Boolean	Biyani et al., 2016 [57]

For example, the post text “It’s another retail milestone on the road to Christmas, and major brands are expecting bumper sales day on Cyber Monday, traditionally seen as one of the busiest online shopping days of the year, with online sales totalling £1.1bn last year” with the link headline “Cyber Monday 2017: all the best UK deals in one list” by *The Guardian* one week after publication received 68 reactions, was shared 11 times and got 39 comments. For the headline, 11 words and an average word length of 3.73 were recorded. This headline showed no question, no unusual punctuation, and no unigram but a typical clickbait phrase from the list (i.e., “best”).

Furthermore, we executed a sentiment analysis on the headline and post texts using VADER (Valence Aware Dictionary and sEntiment Reasoner) sentiment for python. We chose the tool based on its reputation in science and its specialization on social media [77–80]. With this, we can check for negativity bias and provider further insides to the dataset. The resulting values range form -1.00 (very negative) to +1.00 (very positive).

### 4.3 Controlling for topics

As the posts’ topics were expected to influence the degree of interaction, we included them as controls [18]. We sorted the posts in different categories using a self-developed dictionary because other approaches did not work sufficiently (e.g., topics in URLs). We implemented the distinct categories and the respective keywords in an iterative process. We read 500 headlines to gain insights into topics and suitable keywords which we then used to assign all posts to a certain category. After the first automated annotation, we manually checked a random sample of posts that could not be grouped in a category to find additional keywords. We aimed for distinct categories and initially left headlines that could belong to more than one category unassigned. Finally, the script could categorize 65% of all posts (2,865 out of 4,410 posts) according to nine distinct categories that we additionally labeled as soft and hard news in Table 4 [81]. To further improve the coding quality, we then manually assigned the categories to the remainder of 1,545 posts, resulting in a final number of 48 posts that are assigned the category “other”, accounting for 1.1% of all posts in the dataset.

**Table 4:** Categories identified, example keywords and share.

	Categories	Example keywords	Share
Soft news	Media & Celebrities	meghan markle, grammy, actor	24.4%
	Lifestyle	christmas, gift, diet, health	23.7%
	Sports	football, league, world cup	2.8%
	Travel	cruise, hotel, ferry	1.9%
Hard news	Politics	brexit, government, trump	17.6%
	Crime & Law	assault, criminal, murder	13.4%
	Business & Economics	bank, bitcoin, finance	10.1%
	Education & Science	college, university, study	3.9%
	Environment	climate, pollution, fracking	1.1%

A reliability check with two raters of 100 randomly selected posts for the script based approach resulted in a Fleiss’ κ coefficient of .65, which attests to substantial inter-rater agreement [82]. The greatest differences could be found regarding related topics (e.g., politics and economics). The reliability check for 318 random posts of the manual coding resulted in a coefficient of .66.

### 4.4 Statistical analysis

Negative binomial regressions built the core of the analysis and predicted the number of reactions, shares and comments based on different clickbait features. We chose generalized linear modeling because it does not assume normal distribution of residuals. In particular, the negative binomial regression is suitable for over-dispersed count data and describes probabilities of the occurrence of whole numbers greater than or equal to 0 [83,84]. Furthermore, we used the log link function for the dependent count variables; that is, the independent variables were related linearly to the logarithm of the dependent variable. This allowed us to interpret the results in percent changes rather than absolute gains. Equally, we log-transformed the variable *word count* with base 2 to interpret a percent change in the word count with a percent change in the dependent variable.

We included the posts’ topics as controlling factors. As the variables are of binary nature, the reported effects must be interpreted in comparison to its corresponding zero value. The category “other” was not reported in the result table as there is a limited benefit to this. In addition, the news sources’ origin (the United States or the United Kingdom) and the log-transformed number of page followers were included. Thus, we controlled for country-specific differences and various user bases in the interaction with Facebook and news sources. We calculated the main effects models using SPSS 25. We also estimated the dispersion parameter and fixed the scale parameter (1).

## 5. Results

We report the descriptive statistics of the collected and processed posts (Table 5). Each post record in the dataset included information about the site followers, reactions, shares and comments (i.e., meta data), and the annotation results for the headline (from the referenced website) and for the post text. For the dichotomized variables, we report the total occurrences. Log-transformation requires values greater than zero, excluding a few cases for the regression analyses. These cases did not have a post text at all which can happen when the link is copied to the post and then removed once Facebook has built a preview with the headline from the linked source. Similarly, 36 headlines were missing when a video was posted directly on Facebook without a title manually added.

**Table 5:** Descriptive statistics of variables (Occ. = Occurrences, Medn. = Median, IQR = Interquartile Range).

		N	Occ.	Min	Max	Mean	SD	Medn.	IQR
Meta data	Site follower (log_2_)	4410	-	21.42	23.81	22.713	0.817	22.513	1.73
	Reactions (Likes, Haha, …)	4410	-	6	272639	1595.906	6725.427	382.000	999.25
	Shares	4410	-	0	230316	415.260	4443.244	48.000	152.00
	Comments	4410	-	0	147212	434.020	3368.464	105.000	261.00
Headline	Word length (avg.)	4375	-	2.92	9.4	5.127	0.847	5.060	1.15
	Word count (log_2_)	4375	-	0	4.52	3.292	0.532	3.323	0.58
	Question count	4375	180	0	2	0.040	0.202	.00	.00
	Unusual punctuation	4375	196	0	1	0.040	0.207	.00	.00
	Common phrases	4375	540	0	1	0.120	0.329	.00	.00
	Unigrams	4375	434	0	1	0.100	0.299	.00	.00
	Sentiment	4375	-	-.94	.91	-.075	.395	.00	.41
Post text	Word length (avg.)	4410	-	0	15	4.778	1.048	4.700	1.06
	Word count (log_2_)	4397	-	0	7.68	3.582	1.167	3.700	1.58
	Question count	4410	302	0	3	0.070	0.282	.00	.00
	Unusual punctuation	4410	199	0	1	0.050	0.208	.00	.00
	Common phrases	4410	519	0	1	0.120	0.322	.00	.00
	Unigrams	4410	422	0	1	0.100	0.294	.00	.00
	Sentiment	4410	-	-.98	.97	-.006	.414	.00	.59

### 5.1 Effects on the reactions count

Starting with the reactions count (including Like, Love, Haha, Wow, Sad and Angry) the regression model is depicted in Table 6. The strongest effect was found for unusual punctuation in the headline which caused 55.0% more reactions compared to no unusual punctuation. However, unusual punctuation in the post text has a statistically significant negative effect (i.e., an 21.5% decrease). There was also a difference between common phrases in the headline (reduced by 25.9%) and those in the post text (no statistically significant effect). The controls had partially statistically significant effects.

**Table 6:** Results of the negative binomial regression model for the reactions count.

	Parameter	B	Sig.	Exp(B)
	(Intercept)	-12.750	.000	0.001
Headline	Word length (avg.)	–0.206	.000	0.814***
	Word count (log_2_)	–0.008	.842	0.992
	Question count	–0.786	.398	0.456
	Unusual punctuation	0.550	.000	1.733***
	Common phrases	–0.259	.000	0.772***
	Unigrams	–0.077	.262	0.926
	Sentiment	0.173	.002	1.189**
Post text	Word length (avg.)	0.040	.034	1.041*
	Word count (log_2_)	0.063	.003	1.065***
	Question count	–0.239	.000	0.787***
	Unusual punctuation	–0.215	.027	0.807*
	Common phrases	0.057	.369	1.059
	Unigrams	0.334	.000	1.396***
	Sentiment	0.053	.300	1.055
Topics	Business	–0.006	.977	0.994
	Crime	0.007	.971	1.007
	Education	–0.344	.105	0.709
	Environment	–0.314	.248	0.730
	Travel	–0.672	.004	0.511**
	Media & Celebrities	0.164	.395	1.178
	Lifestyle	0.081	.675	1.084
	Politics	0.218	.267	1.244
	Sports	0.053	.812	1.054
Controls	Origin United States	0.087	.050	1.091*
	Tabloid	0.623	.000	1.864***
	Site follower (log_2_)	0.881	.000	2.414***
	(Scale)	1		
	(Negative binomial)	1.638		

* p ≤ .05

** p ≤ .01

*** p ≤ .001.

### 5.2 Effects on the share count

In the second regression model for the share count (Table 7), we observed an increased number of significant effects. Again, unusual punctuation in the headline leads to a strong increase in shares (88.9%), whereas this feature in the post text decreased shares by 44.0%. An increase in the average word length by 1 led to 37.4% fewer shares regarding the headline, but to 6.1% more shares for the post text. The log-transformed variables can be read as the doubling of the post text word count led to 16.7% more shares.

**Table 7:** Results of the negative binomial regression model for the share count.

	Parameter	B	Sig.	Exp(B)
	(Intercept)	–13.024	.000	2.206E-06
Headline	Word length (avg.)	–0.374	.000	0.688***
	Word count (log_2_)	–0.079	.131	0.924
	Question count	–0.405	.001	0.667***
	Unusual punctuation	0.889	.000	2.432***
	Common phrases	–0.304	.000	0.738***
	Unigrams	–0.254	.003	0.776**
	Sentiment	0.105	.133	1.111
Post text	Word length (avg.)	0.061	.011	1.063*
	Word count (log_2_)	0.167	.000	1.182***
	Question count	-0.808	.018	0.446*
	Unusual punctuation	–0.440	.000	0.644***
	Common phrases	0.413	.000	1.512***
	Unigrams	0.240	.006	1.272**
	Sentiment	0.039	.551	1.040
Topics	Business	-0.053	.542	0.949
	Crime	–0.443	.000	0.642***
	Education	–0.466	.000	0.627***
	Environment	–0.548	.059	0.547
	Travel	–1.196	.000	0.302***
	Media & Celebrities	–0.666	.000	0.514***
	Lifestyle	–0.397	.000	0.612***
	Politics	–0.107	.108	0.902
	Sports	–0.043	.769	0.957
Controls	Origin United States	–0.047	.380	0.954
	Tabloid	0.825	.000	2.283***
	Site follower (log_2_)	0.868	.000	2.375***
	(Scale)	1		
	(Negative binomial)	2.375		

* p ≤ .05

** p ≤ .01

*** p ≤ .001.

### 5.3 Effects on the comments count

The model for the comments count entailed several statistically significant effects of clickbait features on the number of comments (Table 8). Four of the six headline features led to a decrease in comments (e.g., common phrases and unigrams). Instead, the unigram feature in the post text was associated with a 27.4% increase.

**Table 8:** Results of the negative binomial regression model for the comment count.

	Parameter	B	Sig.	Exp(B)
	(Intercept)	–6.220	.000	0.002
Headline	Word length (avg.)	–0.199	.000	0.820***
	Word count (log_2_)	–0.237	.000	0.789***
	Question count	-0.033	.763	0.968
	Unusual punctuation	0.829	.000	2.292***
	Common phrases	–0.242	.000	0.785***
	Unigrams	–0.228	.002	0.796**
	Sentiment	0.063	.309	1.065
Post text	Word length (avg.)	0.059	.010	1.061**
	Word count (log_2_)	0.105	.000	1.111***
	Question count	–0.109	.727	0.896
	Unusual punctuation	–0.068	.542	0.935
	Common phrases	–0.034	.628	0.967
	Unigrams	0.274	.000	1.315***
	Sentiment	-0.121	.036	0.886*
Topics	Business	-0.253	.003	0.777**
	Crime	–0.417	.000	0.659***
	Education	–0.766	.000	0.465***
	Environment	–0.631	.004	0.532**
	Travel	–0.886	.000	0.412***
	Media & Celebrities	–0.602	.000	0.548***
	Lifestyle	–0.075	.333	1.078
	Politics	0.247	.001	1.243***
	Sports	0.392	.006	1.481**
Controls	Origin United States	–0.187	.000	0.829***
	Tabloid	0.752	.000	2.121***
	Site follower (log_2_)	0.575	.000	1.777***
	(Scale)	1		
	(Negative binomial)	1.935		

* p ≤ .05

** p ≤ .01

*** p ≤ .001.

## 6. Discussion

The research goal of this study was to examine the influence of clickbait features in Facebook posts on user interaction. In an extension of previous studies on clickbait, we used not only the referenced articles’ headline but also the Facebook post text.

### 6.1 Clickbait features and facebook interaction

Although certain clickbait features enhance user interaction, not all features scholars have associated with increased click-through rates also increase interaction. Table 9 shows whether the effect was in line with the hypothesis (green), in the opposite direction (yellow) or not statistically significant (n.s.).

**Table 9:** Overview of the hypothesized effects and the actual effects.

Feature	Hypothesized effect		Reactions	Shares	Comments
Average word length	Negative	Headline	Negative	Negative	Negative
		Post text	n.s.	Positive	Positive
Number of words	Positive	Headline	n.s.	n.s.	Negative
		Post text	Positive	Positive	Positive
Contains questions	Positive	Headline	n.s.	Negative	n.s.
		Post text	Negative	Negative	n.s.
Unusual punctuation	Positive	Headline	Positive	Positive	Positive
		Post text	Negative	Negative	n.s.
Typical clickbait phrases	Positive	Headline	Negative	Negative	Negative
		Post text	n.s.	Positive	n.s.
Unigrams	Positive	Headline	n.s.	Negative	Negative
		Post text	Positive	Positive	Positive
Sentiment	Positive	Headline	Positive	n.s	n.s
		Post text	n.s	n.s	Negative

Unusual punctuation in the headline predicted an increase in reactions, shares and comments—up to 2.5 times more than without the punctuation. However, the use of unusual punctuation in the post text either did not show statistical significance or was associated with a decrease in shares. As we expected the opposite direction, the results may indicate that unusual punctuation in post text—in contrast to headlines—disturbs the reading flow or even gives the impression of poor journalistic quality [25]. Regarding the use of questions, the present results indicate a negative effect on the interaction for headlines and post texts, however 50% of the results were not significant, prohibiting a solid hypothesis confirmation. As Biyani et al. (2016) [57] found questions were an important clickbait feature, we also expected questions to evoke interaction, and in particular, comments. Kuiken et al. (2017) [52] hypothesized a positive effect of questions on the click-through rate as well but found the opposite effect for article headlines. Thus, the present data complement the findings of Kuiken et al. (2017) [52], as the results allow the assumption that questions lead to neither more clicks nor more user interaction. Hence, the regularly promoted strategy of asking the audience for opinions should not be deployed by media organizations as it does not contribute to more comments, and even has a negative effect on clicks, reactions and sharing. This could be because headlines that include questions are perceived as providing less adequate information [53]. Thompson, Wang, and Daya (2019) [85] found that users try to avoid sharing high-quality news as they fear damaging their own status, in which the clickbait feature of questions in the headline might lead to a decrease in shares.

Regarding the average word length, the models showed a negative effect of longer words for headlines but showed a positive effect for the post text. As post texts can be longer, once the reader starts reading, the word length may be less influential. Headlines are shorter and should be perceived and understood as fast as possible. Thus, the negative effect on all three dependent variables may be explained by the easier reception of shorter words. The strongest effect was found for the share count. This result contrasts with literature that proposed a positive effect of longer texts for headlines [52,57,58] which led to the hypothesis. However, the analysis showed that doubling the number of headline words (log-transformed with base 2) led to 23.7% fewer comments; the effects on reactions and shares were not statistically significant. The opposite was observed for the post text. All three models yielded a positive effect of the doubling of the word count. However, this effect cannot be generally linear for random lengths but within a suitable range (in the sample between 0 and 205 words). Generally speaking, the post should be concise, that is, as long as necessary but no longer [73]. We argue that longer post texts generate a higher level of interaction, because they offer even users who have not fully read the article the opportunity to interact with it. Thus, longer post texts may involve those users, too, who are just consuming snippets of information instead of the full article as described by Müller et al. (2016).

The common clickbait phrases that most people directly associate with the aim of generating clicks, surprisingly, did not have the expected effect in the analysis of interaction counts. Headlines—more suitable for phrases like *this will blow your mind* than post texts—were statistically significantly associated with a loss of roughly a quarter reactions, shares and comments in comparison to those that had no such phrases. Thus, the present study findings question the assumption of previous results that typical clickbait phrases increase the click-through rate [58]. Lu et al.’s (2018) study on the relationship between clicks and satisfaction of online news consumers may offer an interesting explanation. The authors assumed that clicks alone do not depict fully whether the reader is satisfied with the consumed content. Therefore, the authors divided the news consumption process into three phases: Before-Read, After-Read, and Post-Task Preference. If the quality of the news is low, then the preference for an article declines after it is read and does not yield further interaction. This implies for the present findings that typical clickbait phrases might lead to a rise in the click-through rate, but the articles fail to active further user engagement in the after-read phase. With the data we obtained, we could measure only the influence on the user interaction, not the actual click-through rates.

A similar picture can be drawn for the unigrams used in headlines that showed the unexpected effect of decreasing shares and comments. For post texts, the models showed a statistically significant positive effect of unigrams on all dependent variables. The reason why our hypothesis that unigrams used in the headline would lead to a greater user interaction needed to be rejected, might be linked to the fact that Biyani et al. (2016) [57] did not measure whether a clickbait headline actually had an effect on click-through rates or other dependent variables, but established the word list to identify clickbait headlines.

We further ran a sentiment analysis focused on social media posts as described previously. The primary reason was to check for a negativity bias, which is not the case. Out of six variables in our model (sentiment analysis for headline and post text for each the reaction, share and comment count), only two cases were statistically significant. For post texts, the comment count is negatively influenced by a negative wording of (we observe a 12.1% increase of comments if a negatively toned post text is used). For headlines, the reaction count is influenced in the opposite way. A positive toned headline will increase the reaction count by 17.3%. With this, only two out of six values show an influence of tone, which does not allow a solid statement about the relationship. However, our results indicate a connection and enhance the current state of research [74,75]

Lastly, the topics and the control variables were varying a lot for all user engagement types. Naturally, tabloid media and sites with many followers also experienced significant increases in all user engagement types. The categories did not play a significant role (expect travel) for the reactions count. However, we observed a decrease for most categories in the share count which was independent from the above-mentioned categorization in hard and soft news. Interestingly, the comment engagement does not decrease as strong for the hard topics as for soft categories (excecption: sports). Politics increase the comment count and business or crime will have not such a negative effect (-25.3% and -41.8% respectively) as soft categories (e.g., media & celebrity -60.2%).

### 6.2 The role of digital nudging in the context of clickbait

With answering the research question, we contributed to the perception of user engagement in the context of clickbait in social media. As motivated in the introduction, the usage of clickbait in the context of quality journalism is seen as skeptical while at the same time digital nudging is said to act in the user’s best interest. The following discussion will elaborate on this controversy and contribute with novel perspectives.

The idea of nudging is to alter the choice architecture when there are good reasons to believe the decision-maker is not able to decide in her or his best interest or when societal goals exceed individual preferences [28]. Regarding news consumption, a simplified presentation of news with entertaining factors has been found to address a different audience which can help to inform this less-interested audience about discourses [86]. Using social media has enabled the spread of news to a broader audience. However, hard news about politics, international affairs or business matters are shared less frequently on Facebook and thus, consumed less [18].

Building a bridge to nudging, the framing of information, that is, a different representation of the same information, is among the potential nudging elements in digital environments [60]. The knowledge that a specific framing, for example, using shorter words for the headline, increases the interaction has been described as a clickbait strategy [52] but can also be used for hard news that otherwise do not receive much attention. Nudging a user towards relevant (hard) news items would not mean coercion, as the individual can still decide to not consider the changed posts. By mimicking the framing of successful posts, this decision against a topic becomes either more conscious or equally unconscious like for topics that are currently successful. We argue that this aspect is a shared characteristic of both digital nudging and clickbait, enabling the application of a nudging perspective on clickbait. Abstracting from the differentiation between soft and hard news and the topic-dependent attractiveness, our control variables did not influence the reactions as much as other researchers have suggested [18]. We hence suggest considering nudge theory as a new perspective on clickbait. The morally reprehensible actions of some outlets to force the audience to click on headlines or links may be justified. The first application of nudging for news content in social media showed how the highlighting or dimming of user interface elements can help improve the assessment of news credibility [68]. This alters the display of the same information as clickbait. The use of clickbait features to nudge users to hard news would be less transparent than to mark credible sources [6]. However, our suggestions are similar to the usual marketing-oriented clickbait. Furthermore, the central characteristics of a digital nudge are prevailing, being the freedom of choice and the action in good faith. This means that users are able to not click on a post with hard news, keeping the last decision with the user, and the nudge will help the user towards a better decision, following the assumption that every user should also be up to date with hard news.

In addition, it would enable editorial teams to tweak their hard news posts, while more transparent changes in user interfaces would require the user to install browser plugins or platform providers to make decisions for the user, for example, to highlight credible sources. Considering our findings with the difference between hard and soft news, we propose the usage of textual framing and thus digital nudge elements independent of the news source.

## 7. Theoretical and practical contribution

The present empirical study on the actual impact of clickbait features on social networks contributes to theory as most scientific studies have examined clickbait features in news articles and their headlines without considering the increasing influence of social media and their interaction capabilities. The regressions yielded novel results that can only partly confirm previous studies outside social media and contributed new findings about the efficacy of clickbait on Facebook and the role of the post text. To the best of our knowledge, this is the first study to examine features known from clickbait in post texts.

Generally, we applied different methodological approaches and set another focus, that is, the interaction between different research scenarios. Biyani et al. (2016) [57] and Chakraborty et al. (2016) [58] used machine learning approaches for feature annotation, but the present study was lexica- und rule-based. Furthermore, Chakraborty et al. (2016) [58] tried to inductively derive clickbait features by comparing the linguistic differences between a priori defined clickbait and a non-clickbait data set. That was different from this study, in which we deductively used predefined clickbait features for the analysis. The third main source for the present study hypotheses (Kuiken et al. (2017) [52]) looked at the influence of clickbait features in headlines on the increase in click-through rates and took the newsletter of the online news kiosk “Blendle” as the object of study. People who subscribe to the newsletter of an online news kiosk are likely to be interested in news in general, whereas users of social media sites like Facebook might be confronted with news serendipitously, while scrolling through their news feed.

We further contribute to theory by discussing the role of digital nudging in the context of clickbait. With the knowledge of nudge elements and characteristics, we argue that the usage of clickbait to convince users towards the fulfillment of their social responsibility (e.g., by reading certain hard news) can be seen as a digital nudge. We do acknowledge boundaries to this argument, as clickbait in general might not act in good faith, but rather in the interest of the outlet. Yet, the use-case we describe complies with the character of a digital nudge. We call for further research to pick up on this argument and validate the assumption for example with an experiment that nudges in exactly this context.

Besides the novelty of our approach and hence the unique theoretical characteristics of the findings, the implications form contributions for practitioners in organizations as well. Editors that make use of clickbait can potentially increase user interaction by (1) using unusual punctuation in the headline, (2) ensuring a longer post text while (3) keeping the words in the headline to a minimum and (4) making use of typical clickbait phrases. With this, practitioners can increase the efficiency when applying clickbait features to their social media posts. Following our findings, each recommendation above will influence the user interaction in a positive direction, and we did not find interferences. In addition, the context of the post did only matter in few cases of our example (we differentiated between hard and soft news). While this is yet to be validated for other contexts, our results allow the assumption that tabloid as well as serious content is equally subject to the proposed measures above and will have a similar positive effect.

## 8. Conclusion, limitations and further research

We examined the use of persuasive instruments in the user interface for journalistic content on social media. This study builds a basis for HCI researchers and journalists, for which clickbait features might be useful to nudge users to interact with relevant news and which do not directly grab the user’s attention. As nudging must be understood as a positive trigger to make better decisions, using features known from clickbait can motivate users to read articles with greater impact. We discussed the overlap of clickbait and digital nudging and in this context reviewed the current state of theory towards potential harmful influence of clickbait to reputable sources. The melting of tabloid and high-quality journalism due to similar presentation techniques is indeed a potential threat that needs further solid research that yet does not exist. However, this study enabled us to draw first careful conclusions that are novel to the current state of research. Our findings allow unique insights into the characteristics of user engagement triggered by clickbait.

Naturally, there are limitations to our work. There are more clickbait features, which could not be examined in this study. For instance, the use of the second person could be an additional clickbait feature, which should be considered in future studies. One major limitation of this study is that the data set did not include any information about the actual click-through rate. Thus, the results did not measure whether clickbait features led to clicks on an article but whether the existence of clickbait features in headlines and post texts on Facebook lead to increased user interaction with a post. Although the degree of shares, comments and reactions on Facebook showed user engagement, which is an important indicator for involvement with the content, it cannot be equated with an actual click on the article. Like the topic categorization, that we executed in two steps, the detection of common clickbait phrases and the sentiment analysis are influenced by the tool choice. Despite the fact that we applied all to the best of our knowledge, other tools might show other results as they use other algorithms.

Besides these limits, we call for further research based on our findings or the discussion. As pointed out, some clickbait features increased user interactivity and could be used to draw attention to important, but unpopular topics. However, the use of clickbait could be perceived as a form of spam with unwanted side effects not studied here (Potthast et al., 2016). Thus, future studies should investigate whether using clickbait features on articles that count as hard news increases user interaction, or whether negative effects such as a decrease in perceived credibility occur and thus adding to this existing body of research [25]. Especially a longitudinal view on user’s perception of clickbait in the context of high-quality journalism is of major interest. This as well can serve as basis for further advances in the field of digital nudging. In this regard it might be worth studying how clicks and reactions are interrelated as the investigated features might have caused a click and then disappointment with the content [27,57].

Additionally, it would be interesting to know whether the use of clickbait features leads not only to more interaction but also to more people reading the linked articles, which would be important as the goal is to inform users of socially relevant topics. To find out whether articles that use clickbait features help create more revenue, it would be important to measure the click-through rate along with other forms of user interaction.

This work provides novel insights into the user engagement of clickbait on social media and its touch points to digital nudging. The perspective on quality journalism and potential good use cases of clickbait in this context are to encourage other scholars to pick up on this path.
